# The BEACON study: an update to the protocol for a cohort study as part of an evaluation of the effectiveness of smartphone-assisted problem-solving therapy in men who present with intentional self-harm to emergency departments in Ontario

**DOI:** 10.1186/s13063-022-06788-7

**Published:** 2022-10-05

**Authors:** Simon Hatcher, Marnin J. Heisel, Oydeji Ayonrinde, Daniel Corsi, Nicole E. Edgar, Sidney H. Kennedy, Sakina J. Rizvi, Ayal Schaffer, Mark Sinyor

**Affiliations:** 1 Clinical Epidemiology Program, Ottawa Hospital Research Institute, 501 Smyth Road, Ottawa, ON Canada; 2 Department of Psychiatry, University of Ottawa, 5457-1145 Carling Avenue, Ottawa, ON Canada; 3 Department of Mental Health, The Ottawa Hospital, 501 Smyth Road, Ottawa, ON Canada; 4 Departments of Psychiatry and of Epidemiology and Biostatistics, Western University, Parkwood Institute, Mental Health Care Building, London, ON F4-365 Canada; 5 Department of Psychiatry, Lawson Health Research Institute, University of Rochester Medical Center, Rochester, NY USA; 6 Department of Psychiatry, Queen’s University, 752 King Street West, Postal Bag 603, Kingston, ON Canada; 7 Kingston Health Sciences Centre, 76 Stuart Street, Kingston, ON Canada; 8 School of Epidemiology and Public Health, University of Ottawa, 600 Peter Morand Crescent, Ottawa, ON Canada; 9 Centre for Depression & Suicide Studies, St. Michael’s Hospital, 193 Yonge St. Suite 6-001A, Toronto, ON Canada; 10 Department of Psychiatry, University of Toronto, 250 College Street, 8th floor, Toronto, ON Canada; 11 Li Ka Shing Knowledge Institute, St. Michael’s Hospital, 30 Bond Street, Toronto, ON Canada; 12 ASR Suicide and Depression Studies Unit, St. Michael’s Hospital, 193 Yonge St, 6-009, Toronto, ON Canada; 13 Institute of Medical Science, University of Toronto, 1 King’s College Circle, Medical Sciences Building, Room 2374, Toronto, ON Canada; 14 Hurvitz Brain Sciences Research Program, Sunnybrook Research Institute, 2075 Bayview Avenue, Toronto, ON Canada; 15 Department of Psychiatry, Sunnybrook Health Sciences Centre, 2075 Bayview Avenue, Toronto, ON Canada

## Abstract

**Background:**

Men who present to the emergency department (ED) with self-harm are at high risk of dying by suicide, with 2.7% of men dying in the year following their presentation, more than double the rate for women (1.2%). Despite this, care received after an ED visit is highly variable and many are not assessed for psychological needs. Furthermore, the limited psychological care that is available is often not covered by provincial health insurance. Even when referrals for follow-up care are made, engagement rates are low. Previous recommendations to improve engagement include written discharge plans, caring contacts, and focused interventions targeting middle-aged men at elevated risk of dying by suicide. Blended care, the incorporation of technology into traditional care, has also been proposed as a method to increase engagement in and clinical benefits from psychotherapy. This project aims to determine whether the delivery of an evidence based treatment (problem-solving therapy (PST)) is enhanced by the addition of a custom smartphone application (BEACON) compared to usual care. Due to the impact of the COVID-19 pandemic on site participation and the planned implementation, we have made several changes to the study design, primary outcome, and implementation.

**Method:**

We originally proposed a cohort study nested within a larger cluster randomized trial wherein intervention sites would deliver the blended care, and control sites, whose personnel were not aware of their participation, would continue delivering usual care. The cohort study evaluated participant level outcomes as previously described by Hatcher et al. (2020). Due to pandemic-related constraints, our number of participating sites dropped to five potential sites which left the cohort study underpowered. As such, we changed the study design to a multi-site, individual randomized controlled trial (RCT) among the five remaining sites. Participants will be randomized to six sessions of therapy (PST) alone, or to the therapy plus BEACON, and followed up for 6 months. Our primary outcome was changed to evaluate feasibility and acceptability with the aim of designing a definitive RCT. Study implementation was reimagined to allow for completely virtual/online conduct to comply with local COVID-19 and institutional restrictions on in-person activities.

**Conclusion:**

This updated protocol will provide strong results for the planning of a definitive RCT of the blended care intervention in the future, addressing areas of difficulty and concern prior to its implementation. We will evaluate the feasibility of the study intervention, assess recruitment and retention of participants, and address challenges with implementing the protocol. Lastly, we will evaluate the appropriateness of our primary outcome measure and accurately determine a sample size for a definitive RCT.

**Trial registration:**

ClinicalTrials.gov, NCT03473535. Registered on March 22, 2018.

## Update

This update describes amendments to study design, recruitment, implementation, and study outcomes and should be read alongside the previously published protocol [1]. The changes were employed due to the COVID-19 pandemic and the resulting impact on sites’ ability to conduct study activities. Consequently, several sites ultimately withdrew their participation, leaving the original study underpowered. All changes in the protocol have been approved by the Board of Record, the Ottawa Health Sciences Network Research Ethics Board (OHSN-REB) via Clinical Trials Ontario (https://www.ctontario.ca/), a centralized REB platform developed to facilitate multi-site clinical trial review in Ontario, Canada.

## Study design

The original study design was a cohort study nested within a larger pragmatic multicenter pre- and post-design cluster randomized trial [1]. The COVID-19 pandemic resulted in a priority shift away from research at our randomized community sites resulting in their complete drop-out from the trial, leaving the cohort study underpowered with only 5 potential sites. As a result, we changed the design to a pilot individual randomized controlled trial (RCT). Instead of all individuals enrolled at a participating site receiving problem-solving therapy (PST) together with access to the BEACON smartphone application (BEACON), sites will now randomize participants to PST alone or PST + BEACON. The length of participation has also been changed with study follow-up decreased from 12 to 6 months. Visits will now occur as per the updated time and events schedule outlined below in Table 1. Eligibility criteria remains largely unchanged, with only the duration from ED presentation to recruitment extended from 2 to 4 weeks to accommodate potential pandemic-related delays in recruitment.

**Table 1:** Revised time and events schedule

**Measure**	**Time needed to complete measure**	**Baseline and session 1**	**Weeks 2–5**	**6-week follow-up**	**Post-study 3 months and 6 months**
PST session	60 min	X	X	X	
Access to the BEACON application (if applicable)	N/A	X	X	X	X
Demographics, readiness and influence of the media	5 min	X			
CMNI	5–10 min	X			
BSS	5–10 min	X		X	X
PHQ-9	5 min	X		X	X
GAD-7	5 min	X		X	X
PC-PTSD-5	3–5 min	X		X	X
EQ-5D	3–5 min	X		X	X
Experienced meaning in life scale	5–10 min	X		X	X
Multidimensional scale of perceived social support	5–10 min	X		X	X
AUDIT-C	2–3 min	X		X	X
AUDIT* *Only to be administered in the event of a positive screen on the AUDIT-C	3–5 min	X		X	X
DAST-10	3–5 min	X		X	X
TiC-P	5–10 min	X		X	X
SPSI-R:S	5–10 min	X		X	X

## Study outcome

In the original study, we had planned to evaluate change in suicidal ideation as measured on the Beck Scale for Suicide Ideation (BSS) [2–4]; however, the study is no longer powered for efficacy outcomes. In order to inform a future definitive trial, the updated primary outcome examines feasibility and acceptability.

Feasibility and acceptability will be evaluated using the following four indicators: (i) eligibility, recruitment, and retention; (ii) patient use and acceptability of the blended intervention; (iii) the primary outcome measure and sample size for a definitive RCT; and (iv) adherence to the protocol.

i) For eligibility, we will retain screen failure data from those participants who have consented to be in the study to assess the frequency at which each inclusion/exclusion criterion are not met. We will assess recruitment by comparing group level demographics at each hospital of men who presented to the ED with self-harm compared with those enrolled in the study. Lastly, for retention, we will assess the characteristics of those who complete 0–2 sessions, 3–6 sessions but not the 6 month assessments, and those who complete 3–6 sessions and all follow-up assessments.
ii) We will assess patient use of the BEACON application using de-identified usage statistics including number of BEACON presses and red pins activated, as well as any periods of app inactivity (more than 7 days). We will also conduct qualitative interviews with participants to assess the use of the BEACON application and the acceptability of the blended therapy, as well as any other treatments used by the participants.
iii) To inform determination of a primary outcome in a definitive trial, we will measure the severity of suicide ideas at six months as measured by the BSS, as described in the original paper. We will use the change in responses as well as the qualitative interviews to determine whether the BSS is an appropriate outcome measure for the definitive RCT. We will also use the change in responses on the BSS to inform sample size calculations for the larger planned RCT.
iv) We will evaluate any protocol deviations, planned or unplanned, as well as modifications requested by sites for the conduct of the study at their site in their REB submission. We will evaluate site level frequency of completion of a Therapy Adherence Form that is completed by the therapist at each study visit documenting which activities were completed.

With the exception of timing of administration, the secondary outcome measures remain unchanged. In the original protocol, many secondary outcome measures were administered weekly. This has been updated to baseline visit, final PST visit (week 6), and follow-up assessments (week 12 and week 24). The updated timeline and outcome administration is outlined in Table 1.

## Implementation

To accommodate the ongoing work from home orders, we adjusted the delivery of study activities, including delivery of therapy, to allow for completely remote visits. For completion of study visits and therapy sessions, a videoconferencing platform will be used to facilitate “face-to-face” interactions. We also worked with the Ottawa Methods Centre to develop and implement a study Electronic Data Capture System (EDCS) that is capable of randomization and data capture. Upon enrollment of a participant, a study staff member enters the individual’s information into the EDCS, which then randomizes each new participant, assigning a unique identifier and study allocation.

Whereas the original protocol utilized paper-based questionnaires, the updated study uses the EDCS to provide a secure link for participants to complete questionnaires in their own home and at their own pace, a change required to facilitate remote study visits. The EDCS also captures staff entered data such as medications and adverse events. Entries are reviewed regularly by study staff and participants are informed that responses may not be reviewed immediately.

## Recruitment

Recruitment has had to adapt to accommodate institutional restrictions for onsite and in-person activities. In many instances, research staff have not been allowed onsite to complete study activities to decrease the burden on the hospital and reduce the risk of transmission and outbreaks. Additionally, most sites are no longer allowed to leave recruitment materials that would be handled by multiple individuals (such as bookmarks and posters) in waiting areas due to infection prevention policies. As such, we have had to adapt our recruitment approach to accommodate for these changing scenarios. We have allowed sites to develop their own recruitment plans to reflect their own internal practices. As sites are based across Ontario, the implementation of regional restrictions has often meant that there is no consistent process across sites. The source of recruitment has not changed; participants must still have been seen in the ED, but recruitment efforts have been expanded to include scanning of medical records to recruit individuals who have consented to be contacted for research after their discharge from ED and recruitment among inpatient units where patients have been transferred to after their ED presentation.

## Sample size

Sample size has been updated to reflect the change in design and outcomes. Calculating sample size for pilot studies is controversial. Calculations may be based on estimation of important parameters with sufficient precision [5], the likelihood of unforeseen problems [6] or rules of thumb such as 12 participants per group [7], at least 9% of the main trial’s sample size [8], or at least 50 participants [9]. Further, there is a lack of guidance on calculating sample size for pilots of multi-center trials where clustering at the different sites may be a factor. Based on previous rigorous randomized controlled trials of interventions in this population, we expect that in the main trial the effect size will be small and the sample size large. We have designed the pilot to estimate the proportion of patients who would meet our feasibility criteria, using confidence intervals. Based on our experience with previous studies conducted in this population, we estimate that enrolling 100 patients across participating sites would allow us to assess our feasibility outcomes and maximize the chance of identifying unexpected barriers to carrying out a larger trial across multiple centers.

## Randomization

The original protocol was a cluster randomized trial where all sites whose personnel were aware of their involvement would receive access to the intervention. With the update to an individual RCT, the randomization criteria had to be redesigned. The updated randomization for this study will occur with 2:1 (67:33) allocation in favor of the blended therapy model across a maximum of five sites. Given the small sample size, there will be no stratification across sites to ensure an equitable allocation to the conditions.

## Statistical analysis

The statistical analysis plan remains unchanged from the initial protocol, except for removal of an interim analysis.

## Open science

In our original publication, we stated our intent to provide datasets on an open access platform (Open Science Framework; https://osf.io/). However, due to concerns around data sharing and privacy, and differing implementations of regulations between sites, this will no longer be possible. All publications resulting from the study will be available in an Open Access format, and de-identified datasets will be available from the principal investigator on reasonable request at the end of the study.

## Current status of the study

This adapted of the protocol was approved and implemented in January 2021. Three sites have been activated and are enrolling participants. The first participant was enrolled in May 2021, and 34 participants have been enrolled as of July 2022. We will continue recruiting through 2022 with a planned end date of March 2023.

## Data Availability

Not applicable.

## Abbreviations

AUDIT: Alcohol-Use Disorder Identification Test
BDI: Beck Depression Inventory
BSS: Beck Scale for Suicide Ideation
CIHR: Canadian Institutes of Health Research
CMNI: Conformity to Masculine Norms Inventory
C-SSRS: Columbia-Suicide Severity Rating Scale
DAST-10: Drug Abuse Screening Test Short Form 10
ED: Emergency Department
EDCS: Electronic Data Capture System
EMIL: Experienced Meaning in Life Questionnaire
EQ-5D-5L: EuroQol 5 Dimensions (5 levels) Questionnaire
GAD-7: Generalized Anxiety Disorder Questionnaire
MSPSS: Multidimensional Scale of Perceived Social Support Questionnaire
OHSN-REB: Ottawa Health Sciences Network Research Ethics Board
PC-PTSD: Primary Care Post-Traumatic Stress Disorder Screening Questionnaire
PHQ-9: Patient Health Questionnaire
PST: Problem-Solving Therapy
RCT: Randomized Controlled Trial
REB: Research Ethics Board
SPSI-R:S: Social Problem-Solving Inventory-Revised Short Form
tiC-P: Questionnaire on Healthcare Consumption and Productivity losses for patients with a Psychiatric Disorder
